# Assisted Reproduction is Not Associated with Increased Risk of Congenital Head and Neck Defects

**DOI:** 10.7759/cureus.2287

**Published:** 2018-03-07

**Authors:** Colin Neumann, Dane A Thompson, Heidi Thorson, James D Sidman, Brianne B Roby

**Affiliations:** 2 General Surgery, Hofstra Northwell School of Medicine; 3 Minnesota Perinatal Physicians, Allina Health; 4 Otolaryngology Head and Neck Surgery, University of Minnesota; 5 Pediatric Ent and Facial Plastic Surgery, Children's Hospital of Minnesota, St. Paul, USA

**Keywords:** artificial reproduction, congenital head and neck, congenital anomalies

## Abstract

This abstract was presented at the American Academy of Otolaryngology-Head and Neck Surgery Annual Meeting, Orlando, FL, September 2014 with the abstract published (Neumann C, Thompson D, and Sidman J; Assisted reproduction is not associated with increased risk of head and neck defects; Otolaryngology-Head and Neck Surgery; Vol 151, Issue 1, supplement, 2014).

Objectives

- Compare the rate of head and neck anomalies between children conceived via artificial reproductive technology (ART) versus those conceived via natural methods.

- Determine the risk of congenital head and neck abnormalities associated with ART.

Study design

A retrospective chart review cross-sectional study from 2004-2014 of all patients admitted to the neonatal intensive care unit (NICU) at a tertiary pediatric hospital.

Results

A total of 14,857 charts were examined; 2,288 patients were conceived via ART, while 12,569 patients were conceived via natural methods. There were 8,022 males and 6,637 females. There were 40 patients born with defects via ART, while there were 681 patients born with defects via natural conception. The total occurrence of congenital malformations was higher for patients conceived naturally versus those conceived with artificial reproduction (5.41% vs. 1.74%). The odds ratio was 0.31 with a 95% CI of 0.23 to 0.43 and a P-value of < 0.0001; the relative risk of having any one of the head and neck defects with ART was 1.04 with a 95% CI of 1.03 to 1.05 and a P-value < 0.0001.

Conclusion

There appears to be no increased risk of congenital head and neck defects in children conceived via ART versus those conceived naturally.

## Introduction

Assisted reproductive technology (ART) is the technology that involves handling eggs, sperm, or both outside the human body (in vitro) in order to achieve pregnancy [1]. ART includes in vitro fertilization (IVF) and can encompass a number of different IVF techniques. ART, in addition to IVF, includes the use of donor eggs or embryos, surrogacy, and intrauterine insemination and ovarian stimulation with gonadotropin or ovarian stimulating medications [1].

Infertility is described as the inability to conceive after one year of attempting pregnancy. Infertility has been identified as a significant independent predictor of adverse obstetrical and perinatal outcomes [2]. Maternal age and obesity are two major factors related to both an increased risk of infertility and an increased risk of adverse obstetrical outcomes. Infertility is associated with male factor or abnormal sperm parameters in approximately 50% of cases [3]. Studies have shown that 4.6% of oligozoospermic men and 13.7% of azoospermic men have constitutional chromosomal abnormalities, the most common being sex chromosomal abnormalities and autosomal translocations [4]. As expected, infertile men with chromosomal abnormalities are more likely to have genetically abnormal spermatozoa and to father chromosomally abnormal pregnancies [5]. Due to the association of infertility and birth defects, there has been some question as to whether it is the inherent risk or the techniques used in ART that cause a higher rate of birth defects or whether there is a combined risk from both factors.

ART, including IVF, currently accounts for 1.7% to 4% of pregnancies [6]. Both primary and secondary infertility can be treated with ART [7]. Examples of situations where ART is used include fertility preservation in patients who have had chemotherapy or other gonadotoxic treatments [8], singles or same-sex couples who want to conceive biologic children, and surrogacy or gestational carriers that will carry biologic children of couples otherwise unable to carry a child during pregnancy [9]. Many ethical issues surround ART and are regulated differently by laws and policies in different countries [10].

There is conflicting evidence for an association between an increased birth defect prevalence and the use of ART although large, population-based studies are scarce [11-13]. This becomes more evident with the recent huge expansion of assisted reproduction techniques involving gamete manipulation, accounting in 2002 for the birth of approximately 223,000 babies worldwide [14]. In some developed countries, the resulting higher birth defect prevalence was found to be 4.2% for ART [12]. Nowadays, concern over the safety of ART pregnancies could be an issue due to interfering iatrogenic factors, such as gamete manipulation, ovulation induction, or luteal phase support drugs, not to mention the underlying causes of infertility [15-16].

While ART techniques have been shown to carry an increased risk for congenital birth defects, no study has attempted to classify the types of births defect. In this study, our aim was to determine if ART methods were associated with a higher risk of congenital birth defects, specifically of the head and neck.

## Materials and methods

After obtaining permission from the Internal Review Board, we conducted a retrospective chart review of all patients admitted to the neonatal intensive care unit (NICU) over a 10- year period from 2004 to 2014 at Children’s Hospital of Minnesota-Minneapolis campus. The patients were split into two groups: patients who were conceived via assisted reproductive techniques and patients who were conceived naturally. Each chart was evaluated for 15 different head and neck malformations: anencephaly, corpus callosum agenesis, Dandy-Walker syndrome, Arnold-Chiari malformations, encephalocele, holoprosencephaly, hydranencephaly, congenital hydrocephalus, tracheal atresia/agenesis, tracheoesophageal fistula, esophageal atresia, cleft palate, facial clefts, Pierre-Robin sequence, and choanal atresia.

Anencephaly, encephalocele, hydranencephaly, and tracheal atresia/agenesis were excluded from the data analysis, as there were no children born from ART who had these malformations. An X2 analysis with a Yates correlation and a two-tailed P value were performed on the total number of anomalies from ART versus the total number of anomalies from natural methods. A multiple linear regression analysis was also performed to account for low birth weight and smaller gestational age.

There was no funding for this study.

## Results

There were a total of 14,857 charts examined; 2,288 patients (15.13%) were conceived via ART, while 12,569 patients (80.02%) were conceived via natural methods. Table 1 shows the patient demographics.

**Table 1:** General Data of Patient Population ᶧ Percentages are # of malformations/total # of natural births ˠ Percentages are # of malformations/total # of ART births ˤ Percentages are # of malformations/total # of all births

	Naturalᶧ	ARTˠ	Totalˤ
Patients	12569	2288	14857
Malformations	681 (5.41%)	40 (1.74%)	721 (4.85%)
Males	6989 (55.61%)	1231 (53.80%)	8220 (55.33%)
Females	5580 (44.39%)	1057 (46.2%)	6637 (44.67%)
Birth Weight (g)	2,556.84 ± 981.67	2,113.42 ± 790.26	2,520.58 ± 967.31
Gestational Age (months)	34.99 ± 4.34	33.43 ± 3.76	34.90 ± 4.27

There were 40 patients (0.27% of the total, 1.75% of ART) born with the aforementioned head and neck malformations via ART, while there were 681 patients (4.58% of the total, 5.42% of natural methods) born with the aforementioned head and neck malformations via natural methods. There were a total of 8,022 males (1,231 ART vs. 6,989 natural) and 6,637 females (1,057 ART vs. 5,580 natural). The average gestational age of all patients was 34.90 ± 4.27 months with an average birth weight of 2,520.58 ± 967.31 grams; the average gestational age of those conceived via ART methods was 33.43 ± 3.76 months with an average birth weight of 2,113.42 ± 790.26 grams; the average gestational age of those conceived via natural methods was 34.99 ± 4.34 months with an average birth weight of 2,556.84 ± 981.67 grams. Using the z-test, there was a statistical significance for both the gestational age average and the birth weight average between the ART and naturally conceived groups with P-value < 0.001. The X2 value with a Yates correlation of a child with a congenital head and neck malformation via ART compared to the natural method was 55.67 with one degree of freedom and a P-value < 0.001. The odds ratio of a child being born with a head and neck malformation via ART compared to the natural method was 0.31 with a 95% CI of 0.23 to 0.43 and a P-value of < 0.0001; the relative risk of the same comparison was 1.04 with a 95% CI of 1.03 to 1.05 and a P-value < 0.0001.

In this study, the total occurrence of congenital malformations in the studied group was higher for patients conceived with natural methods versus those conceived with ART (4.58% vs. 0.27% of the total births). Similarly, in each of the 15 head and neck malformations examined, the rate of occurrence was higher in patients conceived via natural methods versus artificial reproductive techniques. The 11 malformations studied were corpus callosum agenesis (0.41% vs. 0.13%), Dandy-Walker syndrome (0.14% vs. 0.04%), Arnold-Chiari (0.77% vs. 0.22%), congenital hydrocephalus (1.12% vs. 0.31%), tracheoesophageal fistula (0.54% vs. 0.22%), esophageal atresia (0.56% vs. 0.22%), cleft palate (0.96% vs. 0.31%), facial cleft (0.39% vs. 0.17%), Pierre-Robin (0.33% vs. 0.04%), and choanal atresia (0.19% vs. 0.09%). Table 2 shows the different anomalies that were present.

**Table 2:** Number of Congenital Malformations ᶧ Percentages are # of malformations/total # of natural births ˠ Percentages are # of malformations/total # of ART births ˤ Percentages are # of malformations/total # of all births

	Naturalᶧ	ARTˠ	Totalˤ
Corpus Callosum Agenesis	51 (0.41%)	3 (0.13%)	54 (0.36%)
Dandy-Walker Syndrome	19 (0.14%)	1 (0.04%)	20 (0.13%)
Arnold-Chiari Malformation	97 (0.77%)	5 (0.22%)	102 (0.69%)
Congenital Hydrocephalus	142 (1.12%)	7 (0.31%)	149 (1.00%)
Tracheoesophageal Fistula	68 (0.54%)	5 (0.22%)	73 (0.49%)
Esophageal Atresia	71 (0.56%)	5 (0.22%)	76 (0.51%)
Cleft Palate	121 (0.96%)	7 (0.31%)	128 (0.86%)
Facial Cleft	49 (0.39%)	4 (0.17%)	53 (0.36%)
Pierre-Robin Sequence	41 (0.33%)	1 (0.04%)	42 (0.28%)
Choanal Atresia	24 (0.19%)	2 (0.09%)	26 (0.17%)
Total	683 (5.43%)	40 (1.74%)	723 (4.87%)

## Discussion

Controlled studies of perinatal outcomes comparing twin pregnancies conceived after ART with those of spontaneously conceived twins [17], as well as two systematic reviews [18-19], reveal data that are somewhat conflicting. There is data that show a higher risk for major congenital abnormalities in infants following in vitro fertilization (IVF) compared to spontaneous conceptions, even when adjusting for comorbidities and risk factors [20]. A type of IVF called IVF-intracytoplasmic sperm injection (IVF-ICSI) has an increased risk for major malformations of 8.9% in single live births compared with 6.0% in spontaneous conceptions of single gestations and a similar, increased risk in newborns among twin gestations compared to single gestations conceived with IVF-ICSI [21-22].

More recent studies have indicated the complexities of studying infants conceived naturally compared to ART, with some studies showing ART with fewer complications. A study by Joy et al. showed that spontaneously conceived twins had an increase in preterm birth, lower birth weight, and congenital anomalies but the differences were not significant when the monochorionic twin population was removed [23]. Another study by Morcel et al. found that ART resulted in lower-risk pregnancies compared to pregnancies conceived by ovulation induction (OI) [24]. There have been studies showing that subfertility, and not ART itself, actually has a bigger influence on the adverse outcomes in patients undergoing ART [25]. The risk of significant neurodevelopmental or behavioral adverse outcomes at two-year [26] or five-year [27-28] follow-ups appears to be minimal. A 10-year follow-up study found no significant difference between ICSI and spontaneously conceived children [29]. Physical health appears equal, with the exception of increased undescended testes resulting in urogenital surgery in boys conceived by ICSI [30].

The data in this study agrees with the above data in that we did not find a higher occurrence of head and neck defects with ART conception when compared to natural conception. While our study did not separate the various forms of ART, it does provide a large sample size over a decade, which we feel gives an appropriate representation. One point in our study that should be noted is that we only examined children admitted to the NICU, thus automatically selecting sicker, more complicated children. We chose to exclude those patients not admitted to the NICU, as we were intentionally looking for significant birth defects and had we examined all children born to the hospital over this time period, we felt that it would have been a fruitless endeavor. Finally, we did not examine whether or not multiple gestations had an influence on the presence of birth defects in this study.

## Conclusions

Historically, ART has been associated with higher rates of birth defects when compared to natural conception. As ART is becoming more common with newer technologies, newer data is beginning to suggest that the risks are not as high as initially thought. According to this data, there is no increased risk of head and neck defects associated with ART. Further studies are needed, though, to examine the subsets of ART and their independent risks associated with birth defects, specifically of the head and neck.

## References

[1] Walker MEL (2014). Measurement assisted human reproduction outcomes in Canada. A discussion paper prepared for participants of the 2010 Outcomes Roundtable. Government of Canada Publications.

[2] Joffe M, Li Z (1994). Association of time to pregnancy and the outcome of pregnancy. Fertil Steril.

[3] Palermo GD, Neri QV, Monahan D, Kocent J, Rosenwaks Z (2012). Development and current applications of assisted fertilization. Fertil Steril.

[4] Johnson JA, Tough S (2012). SOGC Genetics Committee. Delayed child-bearing. J Obstet Gynaecol Can.

[5] Shi Q, Martin RH (2001). Aneuploidy in human spermatozoa: FISH analysis in men with constitutional chromosomal abnormalities, and in infertile men. Reproduction.

[6] Talaulikar VS, Arulkumaran S (2012). Reproductive outcomes after assisted conception. Obstet Gynecol Surv.

[7] Harper J, Geraedts J, Borry P (2014). Current issues in medically assisted reproduction and genetics in Europe: research, clinical practice, ethics, legal issues and policy. Hum Reprod.

[8] Rodriguez-Wallberg KA, Oktay K (2010). Fertility preservation medicine: options for young adults and children with cancer. J Pediatric Hematol Oncol.

[9] Buckett W (2014). Infertility treatment for non-traditional families. http://www.iaac.ca/en/632-613-infertility-treatment-for-non-traditional-families-by-william-buckett-md-mrcog-fall-2011.

[10] Okun N, Sierra S (2014). Pregnancy outcomes after assisted human reproduction. J Obstet Gynaecol Can.

[11] Gutarra-Vilchez R, Santamariña-Rubio E, Salvador J, Borrell A (2014). Birth defects in medically assisted reproduction pregnancies in the city of Barcelona. Prenat Diagn.

[12] S S, Mayor Mayor (2010). Risk of congenital malformations in children born after assisted reproduction is higher than previously thought. BMJ.

[13] Rimm AA, Katayama AC, Katayama KP (2011). A meta-analysis of the impact of IVF and ICSI on major malformations after adjusting for the effect of subfertility. J Assist Reprod Genet.

[14] Sullivan EA, Zegers-Hochschild F, Mansour R (2013). International Committee for Monitoring Assisted Reproductive Technologies (ICMART) world report: assisted reproductive technology 2004. Hum Reprod.

[15] Halliday J (2012). Outcomes for offspring of men having ICSI for male factor infertility. Asian J Androl.

[16] Zhu JL, Basso O, Obel C, Bille C, Olsen J (2006). Infertility, infertility treatment, and congenital malformations: Danish national birth cohort. BMJ.

[17] Nassar AH, Usta IM, Rechdan JB, Harb TS, Adra AM, Abu-Musa AA (2003). Pregnancy outcome in spontaneous twins versus twins who were conceived through in vitro fertilization. Am J Obstet Gynecol.

[18] Helmerhorst FM, Perquin DA, Donker D, Keirse MJ (2004). Perinatal outcome of singletons and twins after assisted conception: a systematic review of controlled studies. BMJ.

[19] McDonald SD, Murphy K, Beyene J, Ohlsson A (2005). Perinatal outcomes of singleton pregnancies achieved by in vitro fertilization: a systematic review and meta-analysis. J Obstet Gynaecol Can.

[20] Hansen M, Kurinczuk JJ, Bower C, Webb S (2002). The risk of major birth defects after intracytoplasmic sperm injection and in vitro fertilization. N Engl J Med.

[21] Wennerholm UB, Söderström-Anttila V, Bergh C (2009). Children born after cryopreservation of embryos or oocytes: a systematic review of outcome data. Hum Reprod.

[22] Katalinic A, Rösch C, Ludwig M, German ICSI Follow-Up Study Group (2004). Pregnancy and outcome after intracytoplasmic sperm injection: a controlled, prospective cohort study. Fertil Steril.

[23] Joy J, McClure N, Cooke IE (2008). A comparison of spontaneously conceived twins and twins conceived by artificial reproductive technologies. J Obstet Gynaecol.

[24] Morcel K, Lavoue V, Beuchee A, Le Lannou D, Poulain P, Pladys P (2010). Perinatal morbidity and mortality in twin pregnancies with dichorionic placentas following assisted reproductive techniques or ovarian induction alone: a comparative study. Eur J Obstet Gynecol Reprod Biol.

[25] Verstraelen H, Goetgeluk S, Derom C, Vansteelandt S, Derom R, Goetghebeur E, Temmerman M (2005). Preterm birth in twins after subfertility treatment: population based cohort study. BMJ.

[26] Agarwal P, Loh SK, Lim SB, Sriram B, Daniel ML, Yeo SH, Heng D (2005). Two-year neurodevelopmental outcome in children conceived by intracytoplasmic sperm injection: prospective cohort study. BJOG.

[27] Ludwig A, Katalinic A, Thyen U, Sutcliffe AG, Diedrich K, Ludwig M (2009). Neuromotor development and mental health at 5.5 years of age of singletons born at term after intracytoplasmatic sperm injection ICSI: results of a prospective controlled single-blinded study in Germany. Fertil Steril.

[28] Ponjaert-Kristoffersen I, Tjus T, Nekkebroeck J (2004). Psychological follow-up study of 5-year-old ICSI children. Hum Reprod.

[29] Leunens L, Celestin-Westreich S, Bonduelle M, Lieberes I, Ponjaert-Kristoffersen I (2008). Follow-up of cognitive and motor development of 10-year-old singleton children born after ICSI compared with spontaneously conceived children. Hum Reprod.

[30] Ludwig AK, Katalinic A, Thyen U, Sutcliffe AG, Diedrich K, Ludwig M (2009). Physical health at 5.5 years of age of term-born singletons after intracytoplasmic sperm injection: results of a prospective, controlled, single-blinded study. Fertil Steril.

